# Optimal non-pharmaceutical pandemic response strategies depend critically on time horizons and costs

**DOI:** 10.1038/s41598-023-28936-y

**Published:** 2023-02-10

**Authors:** Sarah A. Nowak, Pedro Nascimento de Lima, Raffaele Vardavas

**Affiliations:** 1 Larner College of Medicine at the University of Vermont, Burlington, VT USA; 2 RAND Corporation, Santa Monica, CA USA; 3 Argonne National Laboratory, Lemont, IL USA

**Keywords:** Applied mathematics, Health policy, Computational models, Computational biology and bioinformatics

## Abstract

The COVID-19 pandemic has called for swift action from local governments, which have instated non-pharmaceutical interventions (NPIs) to curb the spread of the disease. The swift implementation of social distancing policies has raised questions about the costs and benefits of strategies that either aim to keep cases as low as possible (suppression) or aim to reach herd immunity quickly (mitigation) to tackle the COVID-19 pandemic. While curbing COVID-19 required blunt instruments, it is unclear whether a less-transmissible and less-deadly emerging pathogen would justify the same response. This paper illuminates this question using a parsimonious transmission model by formulating the social distancing lives vs. livelihoods dilemma as a boundary value problem using calculus of variations. In this setup, society balances the costs and benefits of social distancing contingent on the costs of reducing transmission relative to the burden imposed by the disease. We consider both single-objective and multi-objective formulations of the problem. To the best of our knowledge, our approach is distinct in the sense that strategies emerge from the problem structure rather than being imposed a priori. We find that the relative time-horizon of the pandemic (i.e., the time it takes to develop effective vaccines and treatments) and the relative cost of social distancing influence the choice of the optimal policy. Unsurprisingly, we find that the appropriate policy response depends on these two factors. We discuss the conditions under which each policy archetype (suppression vs. mitigation) appears to be the most appropriate.

## Introduction

The COVID-19 pandemic has called for swift action from public health officials, who have instated Nonpharmaceutical Interventions (NPIs) to curb the spread of SARS-CoV-2 (the virus that causes COVID-19). Because blunt NPIs (i.e., lockdowns) have far-reaching consequences, the question of how society should use the available instruments to curb SARS-CoV-2 transmission has been met with a deluge of research and controversy.

Broadly, two classes of strategies emerged from this debate: suppression and mitigation^1–3^. *Suppression strategies* seek to reduce transmission as much as possible while treatments and/or vaccines are not available through measures such as lockdowns, social distancing mandates, and mask-wearing. Countries known for their strong suppression strategies include New Zealand, Australia, South Asian countries, and others. A *mitigation* (a.k.a. herd immunity, also called “focused protection”) approach attempts to limit the impacts of the disease on the most vulnerable members of the population while allowing the disease to spread, hoping to balance the costs and benefits of social distancing measures^4^. The argument underlying this strategy is that social distancing would be too expensive to contain transmission relative to the cost of infection for the average person. For COVID-19, suppression strategies have deserved and received overwhelming support in the scientific community due to the high death rates caused by the disease, the imminent threat to the stability of health systems and continued uncertainty about the duration of immunity acquired through infection^3,5^. As the COVID-19 pandemic demonstrated, countries that relaxed suppression strategies too soon (e.g., India, Brazil) went through worst-case scenarios where health care was severely impacted, and excess death soared^6,7^.

This paper sits at the intersection of two research fields that have an outsize role in justifying non-pharmaceutical interventions, namely, infectious disease modeling and macroeconomic modeling. Infectious disease models start with a mechanistic description of how a pathogen percolates in society and can be calibrated to data to reproduce the dynamics of a pandemic. During the COVID-19 pandemic, these models have been used to assess tradeoffs associated with pre-specified non-pharmaceutical intervention policies^8–13^. While infectious disease models traditionally do not allow for endogenous behavioral response to the pandemic^14^, infectious disease modelers often account for the most biologically-relevant features of pandemics, including heterogeneity in contact rates and disease severity, and stochasticity in contacts. Infectious disease modelers often focus on model inference using computational-intensive inference approaches. Dynamic macroeconomic models that include infectious diseases also offer a useful framework for evaluating social distancing strategies. Starting from an intertemporal utility maximization problem, dynamic models can be used to demonstrate how a society composed of rational individuals would respond to a pandemic^15,16^.

This paper seeks to contribute to the emerging economic epidemiology modeling field^17^. Prior work in economic epidemiology can be divided into pre-COVID-19 and post-COVID-19 eras. Much of the work pre-COVID-19 focused on vaccination behavior and decisions^14,18,19^, with some analyses of NPI behavior without a central planner.^20^ Since COVID-19, many analyses have examined optimal social distancing behavior and policies in the specific context of COVID-19. Most of these setup problems are solved with dynamic programming methods. Dynamic programming is an exhaustive method for optimization but is also computationally intensive.^21–24^ It requires the definition of discrete time steps, and the solution may depend on this choice. In contrast, we propose a parsimonious model where a central planner seeks to find a time-varying social distancing policy minimizes the costs of social distancing and infection, which can be solved with variational calculus. Variational calculus is a continuous time model formulation that can be more computationally efficient than dynamic programming methods. This enables us to solve the model for a large sample of parameter space. We solve this model over 4212 combinations of model parameters to investigate how the choice of the strategy to govern nonpharmaceutical interventions depends on a set of biological and economic inputs. In our framework, the optimal social distancing strategy depends on the central planner’s assessment of the time-horizon of the pandemic (i.e., the time it takes to obtain and distribute an effective vaccine) and the costs of NPI measures relative to the social cost of infections. We also present a multi-objective formulation that considers trade-offs between the total amount of social distancing and the total proportion of the population infected. The calculus of variations BVP formulation enables us to illuminate the relationship between the single objective and multi-objective formulations and better understand the multiple local minima that arise for some sets of parameters in the single objective formulation.

This paper couples an infectious disease model with a model describing how a central planner would change their social distancing strategies over time. Our analytical structure pairs a deterministic SIR model with a variational calculus approach to optimize a cost function. The dynamic social distancing strategy is defined by the solution to a boundary value optimization problem (BVP). The central planner assumes a final time horizon of the pandemic (e.g., when effective vaccines will be available) and relative costs of social distancing (e.g., the cost of social distancing relative to the burden of the disease).While this framework is not meant to capture the complexity of heterogeneous human behaviors or the stochasticity of epidemics, it provides insight into a central planner’s optimal strategy. We show later that strategies that could be interpreted as suppression and mitigation strategies emerge from this simple model. There is a fast switch from mitigation to suppression strategies as the cost of social distancing and the time-horizon of the pandemic decreases.

## Results

In this section, we present results from both single-objective and multi-objective optimizations. We first present results from our single-objective optimization analysis using the baseline parameter values. We then present the main outcomes of the single-objective optimization for a wide range of parameter values. We performed 4212 optimization experiments to assess how the optimal social distancing policy changes due to economic and epidemiological conditions, as represented by a reduced set of model parameters. Finally, we present results from a multi-objective optimization and show the relationship between these results and the single-objective results.

### Mitigation and suppression strategies emerge from different assumptions

**Figure 1:**
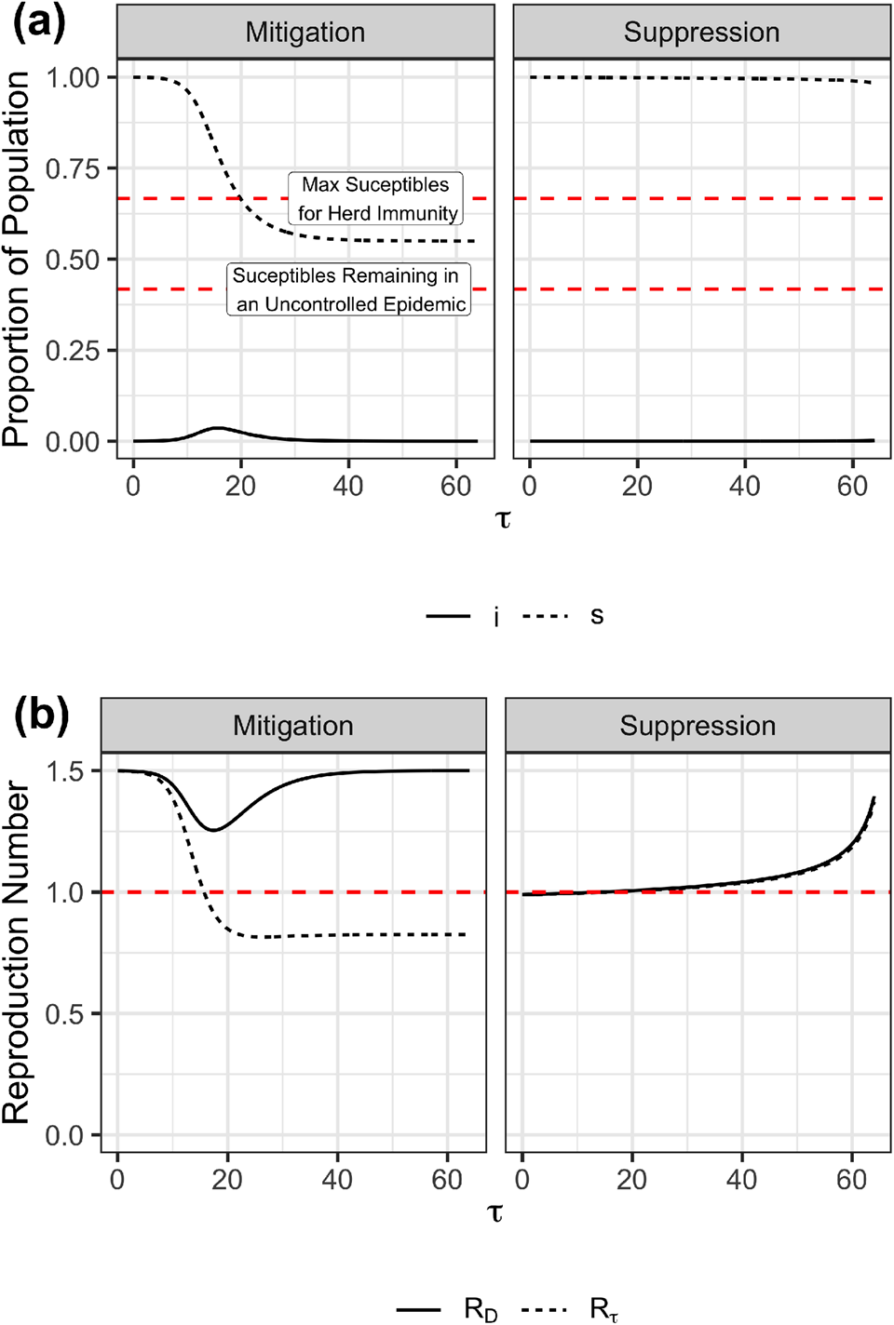
Mitigation vs. Suppression (**a**) The disease dynamics for the proportion of the population infected ($i$) and the proportion of the population susceptible ($s$) are shown. The horizontal line labeled “Max Suceptibles for Herd Immunity” shows $s_H$ and “Susceptibles Remaining in an Uncontrolled Epidemic” indicates $s_{\infty }$. (**b**) The dynamic reproduction number, $R_D$, is shown over time as well as the effective reproduction number, $R_{\tau }$. The horizontal red line indicates where these variables are 1. In all panels, $R_0=1.5$, $i_0 = 10^{-4}$, and $\tau _{{final}} = 64$. The left and right panels in (**a**) and (**b**) differ in the value of $c$, the relative cost of social distancing compared to infections. The left panel shows the qualitative dynamics of a mitigation strategy. In this case $c = 0.4$. The right panel shows the qualitative dynamics of a suppression strategy. In this case $c=0.025$.

Figure 1a presents two different types of dynamics we observe as outcomes of our optimization model, which, for simplicity, we label as “mitigation” and “suppression” strategies. Recall, strategies are not an input to the analysis but an output resulting from the optimization procedure and the underlying assumptions; these two qualitatively different dynamics arise from the model setup. The left panel shows the disease dynamics of a mitigation strategy, and the right shows the dynamics of a suppression strategy. In the mitigation result, the levels of infection peak and then subside, and a substantial portion of the population eventually becomes infected. The mitigation strategy results from a set of parameters representing a relatively high cost of social distancing compared to infection cost. The suppression strategy results from a set of parameters representing a relatively low cost of social distancing compared to infection cost. Therefore, it is unsurprising that the mitigation strategy results in more infections than the suppression strategy. The final proportion of the population infected is less than what would occur if the epidemic spread with no mitigation (i.e., the number of susceptibles remains above the lower dashed red line). In addition, the total number of people who have become infected and have recovered is sufficient to produce herd immunity (i.e., the number of susceptibles is below the upper red line). However, the suppression strategy does not allow the pandemic to take off and therefore results in only a modest number of infections toward the end of the time horizon.

Figure 1b presents the related dynamics of the time-varying reproductive number $R_D$ and the effective reproductive number $R_{\tau }$ as a function of time for both mitigation and a suppression strategy case. The mitigation strategy results in a dynamic reproduction number $R_D(\tau )$ systematically above one, reflecting this policy’s unwillingness to control the epidemic, resulting from the relatively high value of *c*, the cost of social distancing compared to the cost of infection. Social distancing is the greatest ($R_D$ is the lowest) approximately when infections peak (see Figure 1a). In contrast, the suppression strategy keeps $R_D$, and $R_{\tau }$ below one during the beginning of the pandemic. However, it ultimately allows $R_D>1$ once infection levels have become very low and once it is close enough to the end of the pandemic ($\tau _{{final}}$) that some growth in infections still results in very few infections throughout the pandemic. These results align closely with the observed behaviors of countries as vaccines became increasingly available.

**Figure 2:**
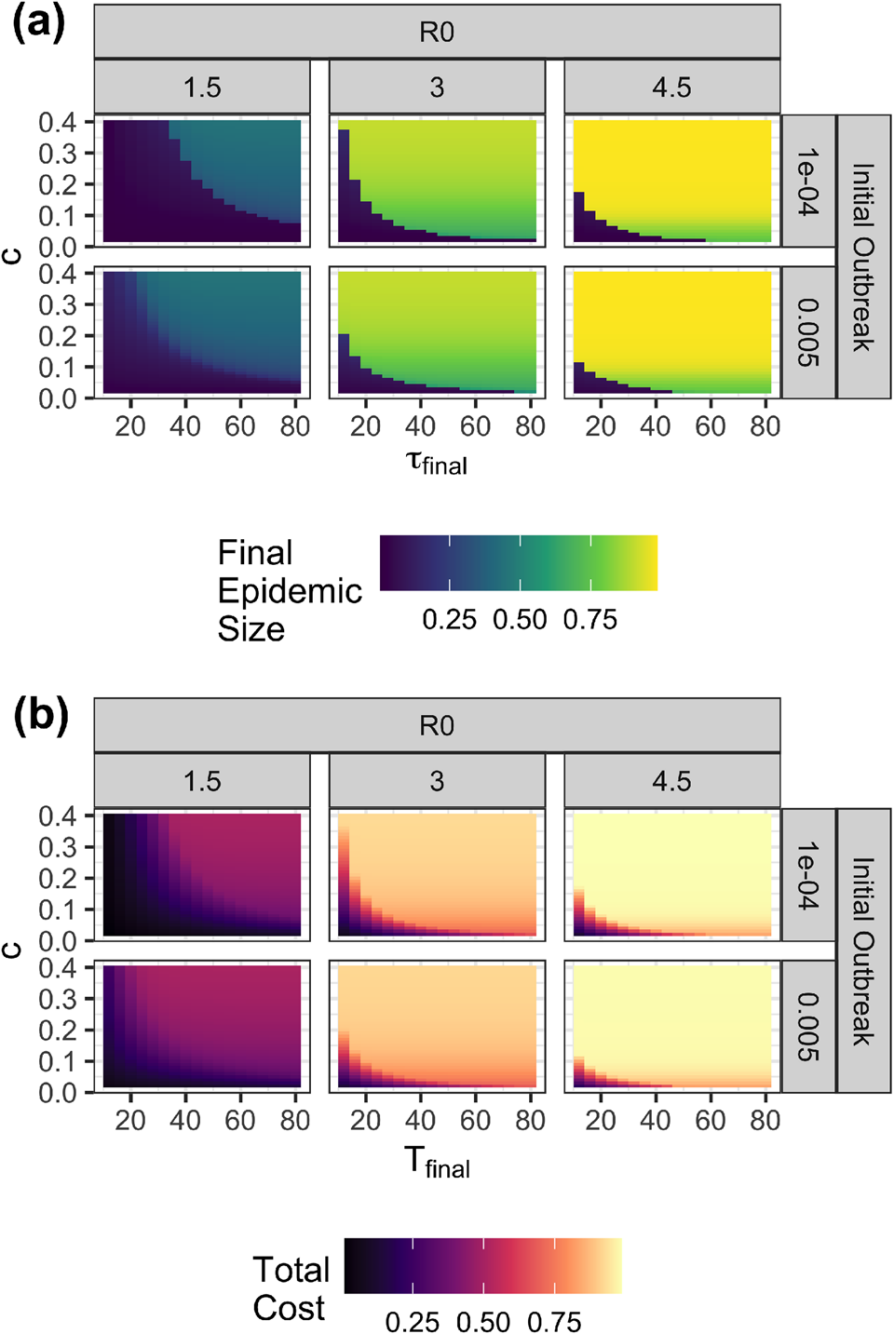
(**a**) Final epidemic sizes and total costs when optimal policies are followed are shown for a range of values of time horizon, $\tau _{\textrm{final}}$ relative cost of social distancing $c$, basic reproduction number $R_0$ and initial outbreak of the size. (**b**) Total costs when optimal policies are followed are shown.

Figure 2a presents the epidemic size resulting from optimal policies when we vary the cost of social distancing *c* and the epidemic duration $\tau _{{final}}$, and perform 4, 212 optimization runs. We report the epidemic size at $\tau _{{final}}$. While we do not impose any artificial policy categories, the figure exhibits a clear discontinuity. The yellow region of the plot, with a long-time frame and high cost of social distancing, results in a relatively large epidemic size and thus reveals a mitigation strategy. Conversely, the central planner chooses a suppression strategy with a short epidemic time frame and/or low relative cost of social distancing. Figure 2b shows the total costs when optimal social distancing strategies are followed. At the boundary between mitigation and suppression strategies, the cost does not change as sharply as the final epidemic size changes. This shows that very different strategies have similar costs near the transition boundary.

**Figure 3:**
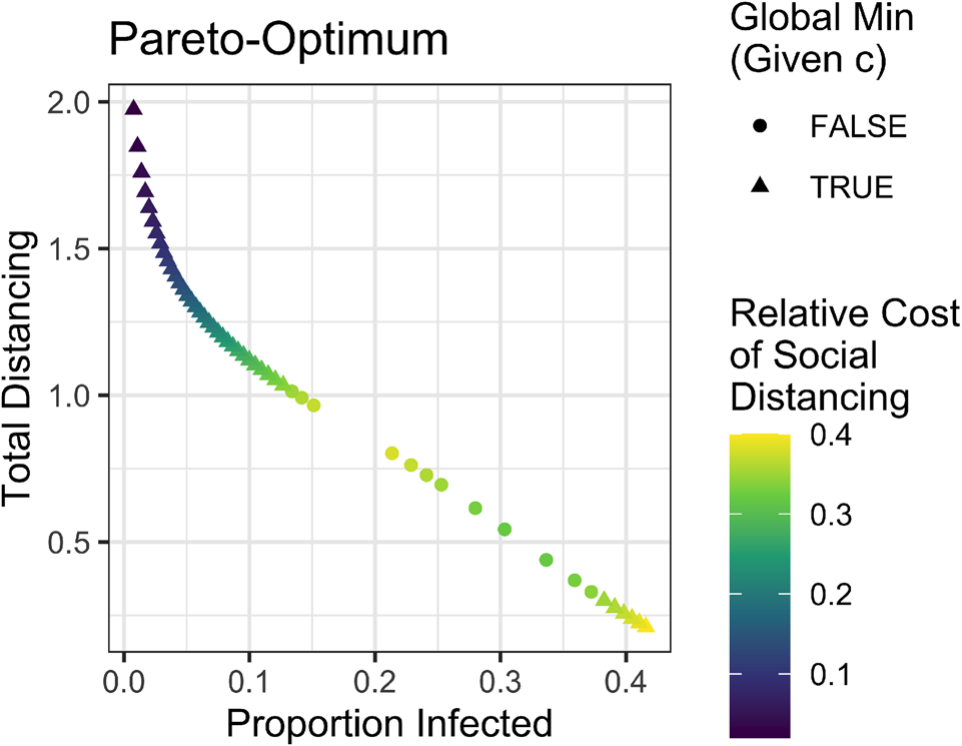
Pareto-optimal multi-objective solutions for $\tau _{{final}} = 36$, $R_0 = 1.5$ and $i_0 = 0.0001$. All multi-objective solutions are mathematically equivalent to local optima from the single-objective formulation. Triangles are points that are equivalent to global optima and circles are points that are equivalent to solutions that are local minima only (not global minima). The points’ colors show the value of $c$ from the single-objective formulation that the solution is equivalent to.

Figure 3 shows the Pareto frontier resulting from a multi-objective optimization. The Pareto frontier contains the non-dominated solutions. These are solutions for which it is impossible to perform better on one metric (infecting fewer individuals or doing less social distancing) without performing worse on the other (doing more social distancing or infecting more individuals). We find that solutions to the multi-objective optimization are mathematically indistinguishable from solutions to the single-objective formulation. (See supplement.) However, many solutions to the multi-objective formulation are local minima only and not global minima of the single-objective optimization (shown as circles in Figure 3). In other words, some solutions are non-dominated, but are at the same time not a local minima for any value of *c*. Solutions on the Pareto frontier that are also global optima to the single objective optimization are those with small or large (but not intermediate) values of the proportion of the population infected, underscoring the dichotomy of the mitigation vs. suppression strategies found in the single-objective formulation.

## Discussion

The results presented revealed the characteristics of optimal social distancing policies under various conditions by formulating the social distancing policy decision as a boundary value problem. In this parsimonious model, a central planner balanced the costs of social distancing against the burden imposed by the disease. To the best of our knowledge, our paper is unique in using a parsimonious calculus of variations approach to examine conditions where a rational central planner would choose a suppression or mitigation strategy. Because this approach does not impose a constraint in the functional form of the solution or impose a discrete time step on which the decisions can be made, social distancing strategies emerge from the problem structure and parameters rather than being imposed a priori. Qualitatively, the results we found in the suppression regime and the mitigation regime are consistent with the findings of prior studies.

**Perceived time-horizons and relative costs shape society’s response to the pandemic**. The most salient finding from the model was that the perceived time-horizon and costs of social distancing provoke a switch from mitigation to a suppression strategy with higher costs of social distancing and a longer epidemic time horizon. This sharp switch from mitigation to suppression might make it challenging for a central planner to maintain the trust of the population in a pandemic with characteristics that fall close to the edge of the frontier revealed in Figure 2a. Because countries with different socioeconomic characteristics and populations with heterogeneous values may perceive or experience social distancing costs differently, any pandemic that falls close to that frontier will inevitably result in disagreement and lack of coordination.

**Optimal strategies are decisive in an idealized setting**. In this mathematical exercise, society’s response to the pandemic was smooth and decisive, meaning that policymakers did not start with blunt lockdowns only to reverse course a few weeks later and instate restrictions again. Therefore, our model does not reproduce policies seen in the real world, including policies that adaptively imposed and lifted social distancing orders. Adaptive policies^25^ and policies with fast periodic switching^26,27^ were tested in our previous work. One possibility is that more nuanced models would reveal situations in which adaptive and periodic policies are optimal and our simplified analytic machinery is not equipped to identify such policies. This aspect should be investigated with further analysis, specifically with more nuanced models that include heterogeneity and stochasticity.

**Completely uncontrolled pandemics are not optimal**. Under the assumptions made in this modeling exercise, completely uncontrolled pandemics were not found to be Pareto optimal - that is, society always did *something* to abate the consequences of the pandemic. While this result is a consequence of our model’s deterministic and continuous nature, it reproduces findings from existing macroeconomic models (see^16^).

**Optimal suppression strategies do not hold** $\mathbf {R_{\tau } < 1}$ **indefinitely**. Early in the COVID-19 pandemic, to disseminate basic concepts of epidemiology to the population, a common view was that society ought to keep COVID-19 cases from growing, and economists have gone a step further to propose that keeping $R_{\tau } < 1$^28^ is necessary. While this seems a reasonable statement, our findings do not corroborate the idea that $R_{\tau }$ should be below one *indefinitely at all costs*. As noted in figures 1a and 1b, $R_{\tau }$ eventually surpasses one before the end of the epidemic time-frame, even under the scenarios that were most favorable to a suppression strategy. These results, therefore, suggest that $R_{\tau } < 1$ is not a permanent end-goal of optimal suppression strategies. Instead, optimal suppression strategies balance the costs and benefits of social distancing measures by keeping $R_{\tau } < 1$ at the beginning of the epidemic and only allow a controlled increase in infections when they are the least dangerous - towards the end of the epidemic. Nevertheless, we did not find an unconditional, permanent suppression strategy optimal from a welfare maximization standpoint using this simple model. Instead, the optimal strategy was contingent and time-varying - it did imply **near** zero-infections at the beginning of the epidemic and then later allowed a modest and controlled rise in infections. This finding also is in line with other studies which found that robust COVID-19 reopening strategies had time-varying reopening thresholds with more stringent thresholds at the start of the pandemic and less stringent thresholds towards the end of the pandemic^25,29^.

**Uncertainty, complexity, and heterogeneity will result in thorny policy tradeoffs.** Based on our findings, one might imagine that a simplified optimization framework such as the one used in this paper will provide a rational approach to support decision-making during the next pandemic. If the costs of social distancing relative to the costs of infection are known and if the time-horizon to a vaccine and the disease parameters are set, and if the model structure is correct, then this framework could provide rational decision support to provide optimal policies. However, we warn the reader that none of these assumptions will hold during the onset of the next pandemic. While the optimization structure used in this paper allows for mathematical tractability, both the costs and the time-horizon of a pandemic are uncertain, heterogeneous quantities. Pandemics will be relatively more costly to societies that cannot mitigate the economic costs of social distancing with income support policies financed by debt.

Similarly, wealthy nations will have access to treatments and vaccines before other nations. And, the costs and benefits of social distancing strategies are not equal for every country^30^. The result is that different countries will be positioned at different regions in the parameter space we presented. This heterogeneity predictably could cause countries to be forced into different strategies. Moreover, within-country dynamics can also play an important role, with different jurisdictions adopting different strategies. The corollary of these factors is that the world *will not* adopt a single best social distancing strategy, and chaotic behavior should be expected. In light of these findings, however, one can still resort to decision-making approaches that seek to find policies that are robust to these uncertain factors but achieve acceptable performance^25^.

While our framework reveals those tradeoffs mathematically, more work is needed to emphasize how policy options could ease those tensions. At a local scale (e.g., state-level decision-making), policymakers can still resort to decision-making approaches that seek to find policies that are robust to the Uncertainty in the global response to the pandemic but achieve acceptable local performance, as demonstrated elsewhere^25^. On a global scale, efforts to expedite the development and global distribution of vaccines can ease the tradeoffs faced by low-income countries.

Although we are not aware of other modeling studies that used the same approach as this paper, our results share similarities with the results found through other modeling paradigms. For example, an existing SIR-macro dynamic modeling study found that optimal containment policies exhibit a sharp initial effort to contain the pandemic in the first few weeks, with a subsequent relaxation^16^. Similarly, a previous study that evaluated a wide range of scenarios found that non-dominated reopening strategies tended to start with stringent reopening thresholds and ended by relaxing interventions towards the end of the pandemic^25^.

## Conclusion

The COVID-19 pandemic has affected billions of people worldwide and has been unprecedented in scale and duration. During the early stages of the pandemic and in the absence of a vaccine, policymakers had to take extraordinary measures, implementing a range of non-pharmaceutical interventions (NPIs) to mitigate deaths caused by the spread of the highly transmissible virus (SARS-CoV-2). These measures included mask-wearing and social distancing policies ranging from partial closings to complete lockdowns.

Despite the deluge of research on COVID-19 policy, the effectiveness of policies as applied to COVID-19 will only partially inform the decision to suppress or mitigate the next pandemic. A new pandemic may bring surprises and different epidemiological characteristics, and the evidence created for COVID-19 might not translate directly to new pathogens with other properties. The intuitive and potentially misleading inclination is to learn what worked for COVID-19 and immediately apply those lessons to a new pandemic. An alternative and complementary learning mode is to understand the fundamental properties of the decision problem we faced in the COVID-19 pandemic and ask how epidemiological and economic parameters should determine our choices in a new pandemic.

As seen in the COVID-19 pandemic, social distancing remains a critical intervention to mitigate the spread and deaths caused by a novel, highly transmissible infectious disease. However, prolonged social distancing can lead to significant declines in social well-being and widespread economic hardships and uncertainties. Under these circumstances, policymakers need to make the discomforting decision of social distancing intervention policies that make a tradeoff between lives and livelihoods.

In this paper, we investigated how the choice of the strategy to govern NPIs changes depending on a central planners’ assessment of the time-horizon of the pandemic (i.e., the time it takes to obtain and distribute an effective vaccine) and the costs of social distancing measures relative to the social cost of infections. In our model, which assumes that society is populated by hyper-rational agents and governed by a rational policymaker, we have found that both mitigation and suppression strategies are possible. Mitigation strategies occur when the central planner believes that social distancing is too costly or if the pandemic would take too long to curb. These results point to the importance of public messaging. They also provide an internally consistent mathematical explanation of why NPIs have enjoyed low popularity and adherence in some world areas.

## Methods

Here we provide a brief outline of our approach. The supplementary methods provide mathematical details that will interest infectious disease modelers and mathematicians. To solve our boundary value optimization problem, we use a variational analysis approach, a mathematical technique used to derive functions that minimize or maximize a quantity, such as a cost over an interval. We combine a standard Susceptible-Infected-Recovered (SIR)^31^ epidemic model describing disease transmission with cost functions describing both costs of infections and costs of social distancing specified over the time horizon of interest.

The state variables of the SIR model are the population densities of the susceptible (*s*), infected (*i*), and recovered (*r*) populations. A key model parameter is the basic reproduction number in the absence of any behavioral change, $R_0$. We solve for a dynamic reproduction number, $R_D$, which is the reproduction number that is modified by social distancing policy that results in behavioral change. The variable $R_{\tau }$ is the effective reproduction number ($R_{\tau } = sR_D$) that captures both the change in the reproduction number due to behavioral changes *and* due to the removal of susceptibles from the population.

In the optimization, we define a total amount of social distancing to be k_SD_, which is given by:

1 $$k_{{SD}} = \int_{0}^{{\tau _{{final}} }} g (R_{D} /R_{0} )d\tau$$

where *g*(*x*) describes a relative intensity of social distancing for a given fractional reduction in the reproduction number ($R_D/R_0$). This function was chosen for simplicity and to enable use of the variational approach to solve the optimization problem. Other methods such as dynamic programming allow discontinuities and imposition of bounds for a solution variable while our variational approach does not. Prior work has often considered a linear function for the cost of social distancing.^24,32^ Our choice of social distancing cost function guarantees that the solution will be appropriately bounded with the variational approach. See the methods supplement for additional information. The total cost of infection is given by the proportion of the population infected is $k_{{\inf ect}}$, which is given by:

2 $$k_{{\inf ect}} = \int_{0}^{{\tau _{{final}} }} {(R_{D} si)} d\tau .$$

Defining a cost of infection by counting the number of infections is common in economic epidemiology^17^.

In both the single-objective and multi-objective optimizations, we model the decision of a central planner who is considering the costs of social distancing and infection over a fixed time horizon without time discounting. First, we formulate the problem as a single-objective optimization model where a cost parameter *c* describes the relative cost of social distancing compared to the cost of infection. We also define $\tau _{{final}}$, which describes the time at which the planner expects the epidemic to end or pharmaceutical interventions become available. The initial prevalence of the disease $i_0$ is the final input. In the single objective formulation, the planner seeks to minimize a total cost, given by:

3 $$Total{\text{ }}\text{Cos} t = ck_{{SD}} + k_{{\inf ect}}$$

We additionally formulate the model as a multi-objective optimization problem in which k_SD_ and $k_{{{\text{inf}}ect}}$ are the two objectives. We seek non-dominated solutions in which k_SD_ cannot be decreased without increasing $k_{{\inf ect}}$ and $k_{{\inf ect}}$ cannot be decreased without increasing k_SD_. Table 1 summarizes the input parameters to our model and provides the values we used in this study.

**Table 1:** Input Parameters and their considered values.

Input parameter	Description	Value or range
$R_0$	The basic reproductive number.	1.5 to 4.5
$i_0$	The initial prevalence expressed as a proportion of the total population.	$10^{-4}-0.005$
*c*	The cost of social distancing expressed as a proportion of the cost of infection.	0.02 to 0.4
$\tau _{\textrm{final}}$	The duration of the fixed time horizon expressed as a proportion of the disease progression time scale $\gamma ^{-1}$.	4 to 80

The estimated value of the basic reproductive rate ($R_0$) for the 2009 influenza pandemic was 1.5, while the estimate for COVID-19 is 2.5 with a range of 1.8 to 3.6^33^. However, during the epidemic’s early stages and in a few countries, the COVID-19 basic reproductive rate has been estimated to be close to 4.5^34,35^. The parameter $\gamma ^{-1}$ represents the expected infectious duration and defines the scale of our time intervals. If we assume that its value is roughly ten days (i.e., similar to COVID-19  ^36,36,37^), we can interpret the final time horizon $\tau _{{final}}$ in Table 1 as ranging roughly between over one month for 4 intervals, to over two years for 80 intervals. Similarly, we can interpret the range of input values we chose for *c* as roughly the inverse length of time of social distancing that is as costly as infection (within an order of magnitude). For the value $c=0.025$, a duration of forty intervals (i.e., $c^{-1}$) of social distancing, which is just over one year, is perceived to be as distressing as the prospect of contracting the disease. For $c=0.4$, a duration of two and a half intervals of social distancing, which is roughly just under a month, is perceived to be as distressing as the prospect of contracting the disease. Due to scaling, we expected that the product of the time horizon $\tau _{{final}}$ and the cost parameter *c* would be critical in determining the transition between suppression and mitigation. Therefore, we chose to use a wide range of values for $\tau _{{final}}$ and *c* to capture the transition boundary between the two strategies, which, based on its interpretation, are plausible.

The parameter $i_0$ provides the initial prevalence expressed as a proportion of the total population at the beginning of intervention measures. Estimates for COVID-19 infections before the beginning of the UK national lockdown in March 2020 were over one hundred thousand^38^. This translates to an $i_0$ value of roughly $1.7\times 10^{-3}$. Our estimate for the range of $i_0$ varies from roughly one-tenth to o three times the case rate estimated in the UK.

### Herd immunity and uncontrolled epidemics

Interpretation of our final results is aided by understanding two conceptually important values of *s*. The first is $s_{\infty }$. This is the proportion of the population that would remain in a susceptible state at the end of an uncontrolled epidemic given that the epidemic started with a low initial infection rate ($i_{0} \ll 1$) that most individuals were susceptible to start (i.e., no individuals were in the recovered state or already immune to the disease at the start of the epidemic). The second important quantity is $s_H$. This is the maximum proportion of individuals who could be in the susceptible state in a population that has achieved herd immunity; if $s_H$ individuals are susceptible and the proportion of the population in the recovered state is $1-s_H$, herd immunity will have been achieved. If a small number of infectious individuals were introduced into the population, the infection rate would decline rather than increase exponentially. For all cases where $R_0>1$, we have that $s_H>s_{\infty }$. In other words, in an uncontrolled epidemic, more people would become infected than would be required to achieve herd immunity.

### Data availability

Code and data generated for the current study are available at https://github.com/pedroliman/covid19-bpv/tree/Scientific-Reports-2022/2023.

## Supplementary Information

Supplementary Information.
