# Supplementary material for: Optimal non-pharmaceutical pandemic response strategies depend critically on time horizons and costs
Source: Sci Rep. 2023 Feb 10;13:2416. doi: 10.1038/s41598-023-28936-y (PMC9912209; doi:10.1038/s41598-023-28936-y)
Supplement: Supplementary file 1 — Supplementary Information. [file 41598_2023_28936_MOESM1_ESM.pdf]

Supplemental Material

Should we Mitigate or Suppress the next Pandemic?

Time-Horizons and Costs Shape Optimal Social Distancing Strategies

Sarah Nowak, Pedro Nascimento de Lima, Raffaele Vardavas

May 13, 2022

1 Methods

1.1 The SIR Model

We use a standard Susceptible, Infectious, Recovered (SIR) model of disease transmission. The standard SIR model describes disease transmission in a well-mixed system in which an interaction between any two individuals is equally likely. Our approach implements the non-dimensional form of the standard SIR model because we couple it to the dimensionless forms of cost functions to find solutions to the social distancing problems. We start by providing a derivation of the non-dimensional SIR form starting from its standard form.

The differential equations describing the dynamics of the susceptible, infectious, and recovered individuals in the population are:

$$\begin{aligned}\frac{d}{dt}S &= -\frac{\beta SI}{N}\\ \frac{d}{dt}I &= \frac{\beta SI}{N} - \gamma I\\ \frac{d}{dt}R &= \gamma I\end{aligned} \tag{1}$$

where S is the number of the susceptible, I is the number of the infectious, R is the number of the recovered, and N is the total number of individuals in the population. The parameters β and γ respectively represent the transmission and recovery rate and have units of inverse time.

1.1.1 Non-Dimensionalization

As is typically done, we define the dimensionless unit of time τ, which is:

$$\tau = \gamma t. \tag{2}$$

In other words, in the dimensionless SIR model, $\tau = 1$ represents the average duration an individual is infectious for. We further recast the state variables in their dimensionless form where

$$\begin{aligned}s &= S/N,\\ i &= I/N,\\ r &= R/N.\end{aligned} \tag{3}$$

The dimensionless differential equations for the population dynamics become:

$$\begin{aligned}\frac{d}{d\tau}s &= -\frac{\beta}{\gamma}si,\\ \frac{d}{d\tau}i &= \frac{\beta}{\gamma}si - i,\\ \frac{d}{d\tau}r &= i.\end{aligned} \tag{4}$$

By defining the reproduction number $R_0 = \frac{\beta}{\gamma}$, these equations can be formulated as

$$\begin{aligned}\frac{d}{d\tau}s &= -R_0 si,\\ \frac{d}{d\tau}i &= R_0 si - i,\\ \frac{d}{d\tau}r &= i.\end{aligned} \tag{5}$$

1.1.2 Final Epidemic Size

Without mitigation measures, and under the assumption that the initial conditions for the proportion of the population infected is very small (i.e., $i(\tau = 0) \ll 1$), and nearly the entire population is initially susceptible (i.e., $s(\tau = 0) \approx 1$), the proportion of the population that remains susceptible as $\tau \to \infty$ is given by the solution to the equation [Diekmann(2013)]

$$s_\infty = e^{-R_0(1-s_\infty)}, \tag{6}$$

and the final epidemic size is $1 - s_\infty$.

1.1.3 The Effective Reproduction Number and Herd Immunity

The effective reproduction number R_τ describes the average number of infections produced by each infected individual in the population at time τ. In the standard SIR model without mitigation measures,

$$R_\tau = sR_0. \tag{7}$$

Herd immunity occurs when the number of those susceptible in the population is reduced either by natural infection or vaccination such that the effective reproduction number is less than 1. Therefore, the maximum proportion of susceptible individuals in a population with herd immunity is

$$s_H = \frac{1}{R_0}. \tag{8}$$

1.2 Time-Dependent Transmission

In classic SIR models, β and R_0 are constant parameters. This paper considers the case where the transmission rate, β, is dynamic and influenced by behavioral changes. We will use the notation that β is a dynamic variable and β_0 is the transmission rate without any behavioral mitigation. Similarly, we define $R_D(\tau)$ as the dynamic reproduction number, which is a function of social distancing behavior. R_0 is the reproduction number in the absence of any behavioral mitigation. In a dynamic framework, the dimensionless SIR model, Equations 5 become:

$$\begin{aligned} \frac{d}{d\tau}s &= -R_D si, \\ \frac{d}{d\tau}i &= R_D si - i, \\ \frac{d}{d\tau}r &= i. \end{aligned} \tag{9}$$

In the framework with a dynamic transmission rate, the effective reproduction number becomes:

$$R_\tau = sR_D. \tag{10}$$

1.3 Cost Functions

We want to find the social distancing policy that minimizes a total cost, which includes the cost of social distancing and the cost of infections over a pre-specified time horizon ranging from time $t = 0$ to $t = t_{\text{final}}$. The social distancing policy is described by $\beta(t)$, the transmission rate, that depends on social distancing behavior at time t. We further define β_0 to be the infection rate for

the disease in the absence of any behavioral change. Therefore, $\beta(t)$ is restricted to the interval $(0, \beta_0]$ The total cost is:

$$\text{Total cost} = (\text{Social distancing cost}) + (\text{Cost of infections}) \tag{11}$$

$$\int_{t=0}^{t_{\text{final}}} H_{\text{total}}(t)dt = \int_{t=0}^{t_{\text{final}}} \left[H_{\text{sd}}(t) + H_{\text{infect}}(t)\right] dt, \tag{12}$$

where $H_{\text{sd}}(t)$ is the cost of social distancing per unit time, and H_{infect} is the cost of infections. As the transmission rate $\beta(t)$ decreases, the cost of social distancing per unit time, $H_{\text{sd}}(t)$, increases, but the cost of infections per unit time $H_{\text{infect}}(t)$ decreases.

1.3.1 Cost of Infections

The number of new infections per unit time is $\frac{\beta SI}{N}$, which comes from the SIR equations. If we define D to be the average cost of infection per infected individual. Then, the cost per unit time of infections is:

$$H_{\text{infect}}(t) = D\frac{\beta(t)S(t)I(t)}{N}. \tag{13}$$

Note that the total cost of infections is:

$$\text{Cost of infections} = D[R(t_{\text{final}}) + I(t_{\text{final}})]. \tag{14}$$

1.3.2 Cost of social distancing

We assume that the total cost of social distancing is proportional to the size of the population N, and a cost parameter C, which has units cost per person per unit time. Therefore, we define the cost per unit time of social distancing to be:

$$H_{\text{sd}}(t) = NCg(\beta(t)/\beta_0). \tag{15}$$

Here, $g(x)$ is the function that defines the relationship between the relative cost of social distancing and relative reduction in the transmission parameter. The function $g(x)$ should have the following properties:

1. **g(x) is a monotonically decreasing function on the interval $[0, 1]$.** The theoretical setup of the problem determines this property. The cost of social distancing should increase as the transmission rate is further decreased.

2. $\lim_{\mathbf{x}\to\mathbf{0}^+} \mathbf{g}(\mathbf{x}) = \infty$. In theoretical terms, it is not possible to stop all transmission completely; therefore, the cost of decreasing transmission rates to zero should be infinite. From a practical perspective, this restriction will prevent optimal solutions to $\beta(t)$ from passing through $\beta(t) = 0$, and if the initial condition for $\beta(t)$ is positive, the solution will remain positive.

3. $\frac{\mathbf{d}}{\mathbf{dx}}\mathbf{g}(\mathbf{x})|_{\mathbf{x}=\mathbf{1}} = \mathbf{0}$. Theoretically, if there is no cost of infections, the optimal $\beta(t) = \beta_0$. Therefore, the cost of social distancing should be minimized when $\frac{\beta(t)}{\beta_0} = 1$. From a practical perspective, combined with condition 2, this condition ensures that solutions to $\beta(t)$ will be bounded to the interval $(0, \beta_0]$ if correctly initialized to the interval.

4. **g(1)=0**. When $\frac{\beta(t)}{\beta_0} = 1$, there are no behavioral changes so the cost of social distancing should be zero.

Based on these three required properties, we choose the function to have the form

$$g(x) = -\ln(x) + x - 1, \tag{16}$$

and thus, substituting this into equation 15, the cost per unit time of social distancing is

$$H_{\text{sd}}(t) = -NC \ln\left(\frac{\beta}{\beta_0}\right) + NC\frac{\beta}{\beta_0} - NC. \tag{17}$$

1.3.3 Dimensionless Cost Equations

We now derive the dimensionless form of the cost equation. We define a dimensionless cost of social distancing, c to be:

$$c = \frac{C}{D\gamma}. \tag{18}$$

Effectively, c compares the cost of social distancing to the cost of infection. Recall that C has dimensions of cost per individual per unit time, D has dimensions of cost per individual, and γ has units of inverse time. Therefore, c is dimensionless. When c is small, people will more willingly social-distance as they feel that the cost of becoming ill outweighs the benefits of mixing socially. Their preference favors their lives and health compared to livelihoods and non-health-related well-being. When c is large, people's preferences are switched. We define the dimensionless cost:

$$h = \frac{H}{DN}. \tag{19}$$

We can make the following substitutions in all equations:

$$\begin{aligned} \frac{\beta_0}{\gamma} &= R_0, \\ \frac{\beta}{\gamma} &= R_D. \end{aligned} \tag{20}$$

Then, the dimensionless cost of infection, Equation 13, becomes:

$$h_{\text{infect}} = R_D s i. \tag{21}$$

The dimensionless cost of social distancing, Equation 15, becomes:

$$h_{\text{SD}} = c g(R_D/R_0). \tag{22}$$

We denote the cost function as $h(s, i, R_D)$ which can be expressed as:

$$h(s, i, R_D) = c g(R_D/R_0) + R_D s i. \tag{23}$$

By setting $\mathcal{T}_{\text{final}} = \gamma T_{\text{final}}$, the total cost in its dimensionless form is

$$\text{Total Cost} = \int_0^{\mathcal{T}_{\text{final}}} \left[c g(R_D/R_0) + R_D s i\right] d\tau. \tag{24}$$

1.4 Full Optimization

Calculus of variations is used to find the functions $y_j(\tau)$ that maximize or minimize

$$\int_0^{\mathcal{T}_{\text{final}}} \mathcal{L}(y_1(\tau), y_2(\tau), ..., y_n(\tau))d\tau. \tag{25}$$

The functions y_j [Weinstock(1974)] that satisfy the following conditions are extrema of the integral in Equation 25:

$$\frac{\partial \mathcal{L}}{\partial y_j} - \frac{d}{d\tau}\left(\frac{\partial \mathcal{L}}{\partial y_i'}\right) = 0, \tag{26}$$

where y_j' indicated the derivative $\frac{dy_j}{d\tau}$.

Our main objective is to solve for R_D that minimizes Equation 23. Because the integrand is a function not only of R_D, but of s and i as well, we introduce the functional Lagrange multipliers λ_i and λ_s to ensure that the conditions of the differential equations governing the infection dynamics (Equations 5) are met. Then, based on the constraints provided by the SIR dynamics, the full Lagrangian becomes:

$$\mathcal{L}(s, i, R_D, \lambda_i, \lambda_s) = h(s, i, R_D) + \lambda_s \left(s' + R_D si\right) + \lambda_i \left(i' - R_D si + i\right) \tag{27}$$

Note that we do not need to include a differential equation constraint for r since the equations for s and i ensure that $r = 1 - s - i$.

We can now apply Equation 26 to all $y_j \in \{s, i, R_D, \lambda_i, \lambda_s\}$.

$y_j = s$ yields:

$$\lambda_s' = R_D i(1 + \lambda_s - \lambda_i) \tag{28}$$

$y_j = i$ yields:

$$\lambda_i' = R_D s(1 + \lambda_s - \lambda_i) + \lambda_i \tag{29}$$

$y_j = R_D$ yields:

$$R_D = \frac{R_0 c}{c + R_0 si(1 + \lambda_s - \lambda_i)} \tag{30}$$

Note that applying $y_j = \lambda_s$ and $y_j = \lambda_i$ to Equation 26 returns the constraint differential equations for s and i from Equation 5. Note that applying $y_j = \lambda_s$ and $y_j = \lambda_i$ to Equation 26 returns the constraint differential equations for s and i from Equation 5.

The differential equations can then be solved as a two-point boundary value problem. For all cases presented in this paper, we set $i(0) = i_0$, $s(0) = 1 - i_0$, $\lambda_s(\mathcal{T}_{\text{final}}) = 0$, and $\lambda_i(\mathcal{T}_{\text{final}}) = 0$. [rv]the $\mathcal{T}$ is not being displayed correctly - anyone know why? We set λ_s and λ_i to be equal to zero at the end point of the interval because $s(\mathcal{T}_{\text{final}})$ and $i(\mathcal{T}_{\text{final}})$ are unconstrained. We solved the 2-point boundary value problems in R using the bvpSolve package.[Mazzia et al.(2014)Mazzia, Cash, and Soetaert]

1.4.1 Multi-Objective Formulation

So far, we have framed our problem as a single-objective optimization. In other words, we optimized the sum of a social distancing and infection costs, where the parameter c defines how a level of social distancing should be weighed against a level of infections. In a Pareto-optimization formulation, we find the solutions $R_D(\tau)$ for which the total social distancing cannot be decreased without

increasing infections and infections cannot be decreased without increasing the social distancing. Mathematically, the total social distancing, which we call k_{SD} is:

$$k_{\text{SD}} = \int_0^{\mathcal{T}_{\text{final}}} g(R_D/R_0)d\tau \tag{31}$$

and the cost of infections, k_{infect} is given by:

$$k_{\text{infect}} = \int_0^{\mathcal{T}_{\text{final}}} R_D s i d\tau. \tag{32}$$

We recover the total cost defined in Eq. 24 though the equation

$$\text{Totalcost} = \text{ck}_{\text{SD}} + \text{k}_{\text{infect}} \tag{33}$$

We know that k_{infect} is the total number of new infections that occur between time $\tau = 0$ and time $\tau = \mathcal{T}_{\text{final}}$, which means that k_{infect} can be defined as:

$$k_{\text{infect}} = 1 - [s(\mathcal{T}_{\text{final}}) - s(0)] \tag{34}$$

The only term in Eq. 34 that depends on $R_D(\tau)$ is $-s(\mathcal{T}_{\text{final}})$. We can therefore formulate the set of Pareto-optimal solutions as those that minimize k_{SD} for each possible value of $s(\mathcal{T}_{\text{final}})$.

The Lagrangian in this formulation is then given by:

$$\mathcal{L}(s, i, R_D, \mu_i, \mu_s) = g(R_D/R_0) + \mu_s(s' + R_D si) + \mu_i(i' - R_D si + i) \tag{35}$$

The variables μ_s and μ_i are Lagrange multipliers in the multi-objective formulation.

We can now apply equation 26 to the variables in Equation 35. Applying $y_j = s$ yields:

$$\mu_s' = R_D i(\mu_s - \mu_i) \tag{36}$$

$y_j = i$ yields:

$$\mu_i' = R_D s(\mu_s - \mu_i) + \mu_i \tag{37}$$

$y_j = R_D$ yields:

$$-\frac{1}{R_D} + \frac{1}{R_0} + \mu_s si - \mu_i si = 0 \tag{38}$$

$$-\frac{R_0}{R_D} + 1 + R_0 si(\mu_s - \mu_i) = 0 \tag{39}$$

$$R_D = \frac{R_0}{1 + R_0 si(\mu_s - \mu_i)} \tag{40}$$

Now, we show that the formulations are equivalent if $\lambda_s = c\mu_s - 1$ and $\lambda_i = c\mu_i$

Starting from Equation 28, we have:

$$c\mu_s' = R_D i(1 + c\mu_s - 1 - c\mu_i) \tag{41}$$

Which becomes:

$$\mu_s' = R_D i(\mu_s - \mu_i) \tag{42}$$

Starting from Equation 29, we have:

$$c\mu_i' = R_D s(1 + c\mu_s - 1 - c\mu_i) + c\mu_i \tag{43}$$

which becomes

$$\mu_i' = R_D s(\mu_s - \mu_i) + \mu_i \tag{44}$$

Starting from Equation 30, we have:

$$R_D = \frac{R_0 c}{c + R_0 si(1 + c\mu_s - 1 - c\mu_i)} \tag{45}$$

which becomes

$$R_D = \frac{R_0}{1 + R_0 si(\mu_s - \mu_i)} \tag{46}$$

In the alternative formulation, $s(\mathcal{T}_{\text{final}}) = s_{\text{final}}$. In this formulation, because s is fixed at the final endpoint, only $\mu_i = 0$. We know that

$$\lambda_s(\mathcal{T}_{\text{final}}) = c\mu_s(\mathcal{T}_{\text{final}}) - 1 \tag{47}$$

We know that $\lambda_s(\mathcal{T}_{\text{final}}) = 0$. We can therefore calculate c if we solve the equations in our second formulation as follows:

$$\mu_s(\mathcal{T}_{\text{final}}) = \frac{1}{c} \tag{48}$$

Contributions

- Sarah Nowak (Ph.D.) led the effort in conceptualizing and formulating the model structure and led the overall modeling effort, including model implementation. Sarah is the corresponding author of this study and can be contacted at: sarah.nowak@med.uvm.edu
- Pedro Nascimento de Lima (MSc) contributed to the model analysis and interpretation and drafting and revising the manuscript.
- Raffaele Vardavas (Ph.D.) contributed to the model formulation, implementation, and interpretation of model outputs and drafting and revising the manuscript.

Acknowledgements

We wish to thank the National Cancer Institute (R21CA157571), and the National Institute of Allergies and Infectious Diseases (R01AI118705 & R01AI160240) for providing support in projects that led to preliminary work and ideas that motivated this project. Dr. Nowak acknowledges support from the Blodwen S. Huber Early Career Green and Gold Professor in Pathology and Laboratory Medicine at The Robert Larner, M.D. College of Medicine.

Disclaimer

The results and conclusions drawn on this working paper have not been peer-reviewed and do not necessarily represent the opinions of the RAND Corporation.

References

[Diekmann(2013)] Heesterbeek Hans Britton Tom. Diekmann, O. *Mathematical Tools for Understanding Infectious Diseases Dynamics*. Princeton University Press, Princeton, 2013.

[Weinstock(1974)] R. Weinstock. *Calculus of Variations: With Applications to Physics and Engineering*. Dover Books on Advanced Mathematics. Dover Publications, 1974. ISBN 978-0-486-63069-4.

[Mazzia et al.(2014)Mazzia, Cash, and Soetaert] Francesca Mazzia, Jeff R Cash, and Karline Soetaert. Solving boundary value problems in the open source software r: package bvpsolve. *Opuscula mathematica*, 34(2), 2014.
